# Expression and structural analysis of human neuroligin 2 and neuroligin 3 implicated in autism spectrum disorders

**DOI:** 10.3389/fendo.2022.1067529

**Published:** 2022-11-21

**Authors:** Zhenzhen Zhang, Mengzhuo Hou, Huaxing Ou, Daping Wang, Zhifang Li, Huawei Zhang, Jianping Lu

**Affiliations:** ^1^ Department of Biomedical Engineering, Southern University of Science and Technology, Shenzhen, China; ^2^ Department of Orthopedics, Shenzhen Intelligent Orthopaedics and Biomedical Innovation Platform, Guangdong Provincial Research Center for Artificial Intelligence and Digital Orthopedic Technology, Shenzhen Second People’s Hospital, The First Affiliated Hospital of Shenzhen University, Shenzhen, China; ^3^ Guangdong Provincial Key Laboratory of Advanced Biomaterials, Southern University of Science and Technology, Shenzhen, China; ^4^ Department of Child and Adolescent Psychiatry, Shenzhen Kangning Hospital, Shenzhen Mental Health Center, Shenzhen, China

**Keywords:** neuroligin 2, neuroligin 3, cryo-EM, autism spectrum disorders, MDGAs

## Abstract

The development of autism spectrum disorders (ASDs) involves both environmental factors such as maternal diabetes and genetic factors such as neuroligins (NLGNs). NLGN2 and NLGN3 are two members of NLGNs with distinct distributions and functions in synapse development and plasticity. The relationship between maternal diabetes and NLGNs, and the distinct working mechanisms of different NLGNs currently remain unclear. Here, we first analyzed the expression levels of NLGN2 and NLGN3 in a streptozotocin-induced ASD mouse model and different brain regions to reveal their differences and similarities. Then, cryogenic electron microscopy (cryo-EM) structures of human NLGN2 and NLGN3 were determined. The overall structures are similar to their homologs in previous reports. However, structural comparisons revealed the relative rotations of two protomers in the homodimers of NLGN2 and NLGN3. Taken together with the previously reported NLGN2–MDGA1 complex, we speculate that the distinct assembly adopted by NLGN2 and NLGN3 may affect their interactions with MDGAs. Our results provide structural insights into the potential distinct mechanisms of NLGN2 and NLGN3 implicated in the development of ASD.

## Introduction

Autism spectrum disorder (ASD) is a disease characterized by neurodevelopmental disorders of the brain, which is mainly recognized by difficulties in emotional and verbal expression, social communication impairment, and a preference for repetitive actions and behaviors (1). Generally, autistic patients have the condition at the age of 2–3, and the patients’ symptoms last during their lifetime (2). The incidence of autism has increased 40-fold at the start of the 21st century (3). The US Centers for Disease Control and Prevention reports that the incidence of autism is more than 2%, with an average of one in 44 children (3). Imaging studies have found that the volume, structure, and connection of autistic brain regions are abnormal, revealing the abnormal development and function of the nervous system in patients with autism (4). Although genetic studies have identified more than 1,000 autism-related mutations, and most of these genes are closely related to synapse development and nervous system function, their molecular mechanisms have not been well-studied yet (5). In recent years, pregnancy factors are increasingly being paid attention to, which is considered to be the main reason for the increase in the prevalence of ASD in the past decade. Recent epidemiological studies have also shown that maternal diabetes increases the risk of autism in the offspring (6–13). Taken together, these findings indicate that the occurrence of autism is the result of the combined effects of genetic and environmental factors (14, 15).

The mutated genes currently found in autism include neuroligin (NLGN), neurexin (NRXN), and shank. They have distinct roles in synapse formation, elimination, maturation, plasticity, and modulation under the influence of the external environment, ultimately affecting the function of synapses and neural circuits (16–18). Neuroligins are postsynaptic membrane cell adhesion molecules that connect presynaptic and postsynaptic neurons, mediate the transmission of signals between synapses, and shape the characteristics of neural networks through specific synaptic functions (19). There are five members of NLGNs in humans (NLGN1, 2, 3, 4X, and 4Y) and four in rodents (NLGN1, NLGN2, NLGN3, and NLGN4) (20, 21). NLGNs have a unique expression pattern. NLGN1 is mainly expressed at excitatory synapses (22), NLGN2 is mainly expressed at inhibitory synapses (23), NLGN3 is expressed at both excitatory and inhibitory synapses (24), and NL4 expression seems to be limited to the glycinergic synapses of retinal glial cells and several other regions of the central nervous system (25). NLGNs belong to single transmembrane proteins, and they all consist of a large extracellular, acetylcholinesterase-like domain (CLD) and a carbohydrate-binding region, following the transmembrane region of *O*-glycosylation helix and the C-terminal intracellular region as postsynaptic density zone (PDZ) recognition component, which mainly binds to postsynaptic target proteins such as Gephyrin and postsynaptic density protein, thus promoting synaptic differentiation and strengthening the stability of synaptic space and the function of transmitting intersynaptic signals (26). The neuroligin family members share 52% sequence identity (26). Among them, the intracellular region was only 31% consistent, while the extracellular sequence and transmembrane domains were 55% and 91%, respectively (27). The differences in the intracellular and extracellular regions make their functions variable.

The ectodomains of NLGNs can form specific cross-synaptic connections with neurexins (NRXNs) of the presynaptic membrane, and these connections can be regulated by the membrane-associated mucin (MAM) domain-containing glycosylphosphatidylinositol anchor (MDGAs) (28). The MDGAs belong to the immunoglobulin-like (Ig) superfamily with six extracellular Ig domains, following a fibronectin type III-like (FNIII) domain and receptor protein tyrosine phosphatase mu (MAM) domain (29). MDGAs have two members, MDGA1 and MDGA2. They share 54% of identities on the sequence. MDGAs have intracellular regions including a glycosylphosphatidylinositol anchor, which requires an association partner when they participate in protein interactions (29, 30). The MDGAs were reported to suppress synapse formation, by competing with NRXNs for NLGN binding (31).

The current study found that the genetic variation of neuroligin and neurexin can explain about 1% of the occurrence of autism (32). The single mutation R451C of NLGN3 is the first single-nucleotide polymorphism associated with ASD (33). Knock-in of *NLGN3* R451C mainly affects the function of GABA inhibitory synapses, leading to the imbalance of neurotransmitter levels in the brain and brain development disorder. As a result, mice have enhanced inhibitory synaptic transmission and accompanying difficulties in social interaction and enhanced spatial learning ability (34). This is consistent with the findings that many ASD mouse models have synaptopathies (35). These models included NRXNs and NLGNs mutations and recently reported MDGA mutations (19, 36). Recently, studies have shown that the binding of MGDA2 to NLGN2 obstructed inhibitory synapse development in an NLGN1-independent manner (37).

Several high-resolution structures of NLGN/NRXN complexes have been obtained (38–40). Different NRXN molecules and NLGN molecules are similar in binding mode, and the binding regions of MDGA1–NLGN overlapped with those of NRXN–NLGN (38, 40). Although the biochemical experiment indicated that MDGA2 has a similar binding affinity to NLGNs as MGDA1 (41), the role of MDGA2 in neuronal synapse development remains highly controversial between the inhibitory and excitatory synapses, and the molecular mechanism is still unclear. In this study, we solved the high-resolution cryogenic electron microscopy (cryo-EM) structures of human NLGN2 and NLGN3. These structures illustrate that NLGNs are highly conserved in a homodimer arrangement. However, the orientation of two protomers from NLGN2 and NLGN3 has a relative rotation with each other, which may influence the interaction with MDGAs. Our findings provide new clues for further in-depth study of the molecular mechanism of neurodevelopmental disorders including ASD.

## Materials and methods

### Gene cloning, protein expression, and purification

The full-length human *NLGN2* and *NLGN3* genes were synthesized by GENERAL BIOL Co. (Chuzhou, China). The extracellular domain of *NLGN2* gene was then subcloned to secretory vector pSecTAG2B with C-terminal Flag-tag and 6xHis-tag. Full-length *NLGN3* gene was further inserted into pCDNA3.1 plasmid containing C-terminal flag tag. Plasmids encoding NLGN2 and NLGN3 were transiently transfected into Expi293F cells (Thermo Scientific, Waltham, MA, USA) with the polyethylenimine (PEI) reagent. After 4–5 days of culture, the cells were harvested by centrifugation. The cell culture supernatants were passed through a 0.22-µm filter, and the cells were lysed by sonication and then loaded on 2 ml of nickel resin. After binding, proteins were eluted in buffer A containing 25 mM of HEPES at pH 7.5, 150 mM of NaCl, and 500 mM of imidazole. NLGNs were further purified with size-exclusion chromatography loading on Superose 6 10/300 column (GE Healthcare, Chicago, IL, USA), which was equilibrated in buffer B (25 mM of HEPES at pH 7.5 and 150 mM of NaCl). Fractions were assessed by sodium dodecyl sulfate–polyacrylamide gel electrophoresis (SDS-PAGE), concentrated, and stored at −80°C.

### Cryogenic electron microscopy sample preparation and data collection

The purified NLGNs were used to prepare cryo-EM grids with a concentration of 0.25 mg ml^−1^ and applied to the holey carbon film (Quantifoil, Großlöbichau, Germany; R1.2/1.3) grids. The grids were blotted for 2.5 s under 100% humidity at 4°C with Vitrobot Mark IV (Thermo Fisher) and plunge-frozen into pre-cooled liquid ethane. The grids were then observed using Titan Krios microscope (Thermo Scientific) operated at 300 kV and equipped with K3 Summit camera (Gatan, Pleasanton, CA, USA) for NLGN2 or K2 Summit camera (Gatan) for NLGN3. Micrographs were recorded with SerialEM under a nominal defocus value ranging from −1.5 to −2.5 μm and nominal magnification of ×130k for NLGN2 and ×165k for NLGN3, corresponding to calibrated pixel size of 0.92 Å pix^−1^ for NLGN2 and 0.842 Å pix^−1^ for NLGN3. A total of 4,021 and 7,984 micrographs were collected for NLGN2 and NLGN3, respectively. A detailed description of the data collection parameters is available in **Supplementary Table 1**.

### Cryogenic electron microscopy data processing

After contrast transfer function (CTF) estimation, motion correction, and particle picking performed by Relion3.1 (42), the particles of NLGN2 were imported into cryoSPARC v3.3.2 for 2D classification, Ab-initio 3D reconstruction, heterogeneous 3D refinement, and non-uniform refinement (43). For NLGN3, all those steps were performed in Relion3.1. The iterative model building and refinement were performed with Coot and Phenix. The 3D figures were then generated with PyMol and Chimera (44–47). The workflow of data processing is available in **Supplementary Figure 1** for NLGN2 and **Supplementary Figure 2** for NLGN3.

### Generation of diabetic offspring

Diabetic WT C57BL/6/J mice were induced by injection of streptozotocin (STZ). Four-week-old female mice were injected daily with 60 mg/kg of STZ (dissolved in Na^+^ citrate buffer) intraperitoneally after an 8-h fasting period. Animals with blood glucose >10 mmol/L were considered positive, while vehicle injection littermates served as control. The females were then caged with proven males, and male offspring were then used for autism-like behavior tests and sacrificed for further experiments.

### Analysis of mRNA levels by real-time quantitative PCR

The total RNA of the mouse brains was extracted by the RNAzol RT reagent (MRC, OH, USA) using total RNA isolation protocol and was reverse transcribed using HiScript III Reverse Transcriptase (Vazyme, Nanjing, China). Each complementary DNA measuring 100 ng was used to measure target genes. All the primers were designed by Primer 3 Plus software (**Supplementary Table 2**). Real-time quantitative PCR was run on LightCycler^®^ 480 Instrument II (Roche, Basel, Switzerland; product no. 05015243001, 384-well) with the Taq Pro Universal SYBR qPCR Master Mix (Vazyme). β-Actin was used as the control for transcript normalization.

### Quantification and statistical analyses

The qPCR and single-cell sequencing data were given as mean ± SEM, and all the experiments were performed at least in quadruplicate unless indicated otherwise. The unpaired Student’s t-test was used to determine the statistical significance of different groups. Statistical significance was determined by Student’s test analysis (*p < 0.05; **p < 0.01; ***p < 0.001; ns, not significant).

## Results and discussion

### Characteristics of NLGN2 and NLGN3 in maternal diabetes-mediated autism spectrum disorder mouse model and different brain regions

**Figure 1:**
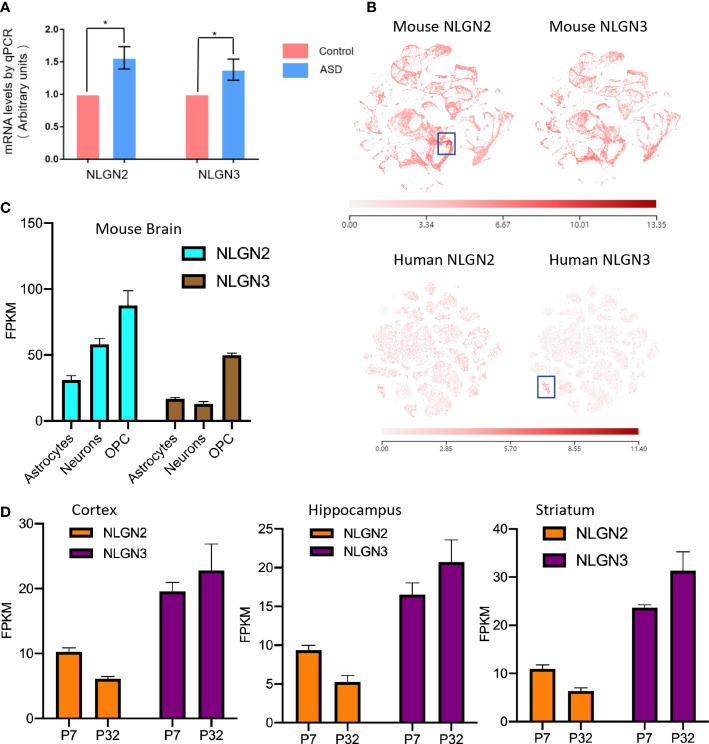
Comparison of NLGN2 and NLGN3 in expression levels and development stage. **(A)** Maternal diabetes induces expression increase of NLGN2 and NLGN3 (*p < 0.05, n = 6) in mouse model. **(B)** The expression levels of NLGN2 and NLGN3 in mouse and human brains by single-cell sequencing. Unit: fragments per kilobase of transcript per million mapped reads (FPKM). **(C)** The expression levels of NLGN2 and NLGN3 in different cells in the mouse brain. **(D)** The expression levels of NLGN2 and NLGN3 in postal 7 and 32 days in cortex, hippocampus, and striatum of mice.

Epidemiological studies have shown that maternal diabetes is closely associated with ASD, and the maternal diabetes mouse model is a well-established model for ASD study (9). To further study the relationship between ASD and NLGNs, we first evaluated mRNA levels in mouse brains from maternal diabetes-induced ASD mice and control mice. The expression levels of NLGN2 and NLGN3 in the ASD mouse group were found to be increased by 56% and 38%, respectively, compared to the control group (**Figure 1A**). This result indicates that the expression levels of NLGN2 and NLGN3 are tightly related to maternal diabetes-induced ASD. We then further examined and compared the expression levels of NLGN2 and NLGN3 based on the deposited single-cell sequencing data from the mice’s cortex and hippocampus (Allen Brain Map database), and we found that the expression pattern of NLGN2 and NLGN3 is similar except for the cortical layer 4 (L4)/5 intratelencephalic cell regions where NLGN2 has a higher expression level (boxed regions in **Figure 1B**, top panel). Further analysis of the single-cell sequencing data of human brain tissue (Allen Brain Map database) shows similar results and further support the conclusion, except for oligodendrocyte progenitor cell (OPC) L1-6 cell regions where human NLGN3 has a higher expression level (boxed regions in **Figure 1B**, bottom panel). Although there are some similarities between NLGN2 and NLGN3 in the overall expression in the brain, we found that the expression of different cells in the mouse and human brain varies. In mouse brain cells, although NLGN2 and NLGN3 have the highest expression levels in OPC, NLGN2 is more expressed in astrocytes than neurons, which is different from that of NLGN3 (**Figure 1C**) (48). This finding was similarly confirmed in the human data (49). Meanwhile, we also found that the increase in the expression levels of NLGN2 and NLGN3 in different brain regions varied in the development stage age (50). NLGN2 shows a higher expression level on postnatal day 7 (P7) compared to that in P32, whereas NLGN3 shows different trends as demonstrated in the cortex, hippocampus, and striatum of mice (**Figure 1D**).

### Determination of cryogenic electron microscopy structures of human NLGN2 and NLGN3

NLGN2 and NLGN3 play different roles in the development of ASD (51), and they have different interactions with MDGAs (24), which are also important in ASD (39). Considering their importance, many studies have been performed to decipher the structural basis of NLGN2 and NLGN3, including the apo form of mouse NLGN2 (52) and mouse NLGN3 (53) and the complex of human NLGN2 and MDGA1 (40). However, the apo form of human NLGN2 and NLGN3 has not been determined yet. This study aims to solve their structures using cryo-EM and compare their differences to deepen our understanding of their distinct roles in the development of ASD.

**Figure 2:**
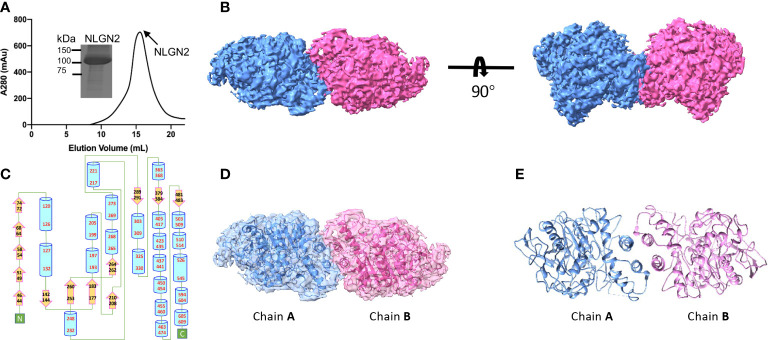
Overall structure of NLGN2. **(A)** Preparation of NLGN2. NLGN2 was expressed in HEK293f cells, purified *via* affinity chromatography and gel filtration, and eluted at about 15.5 ml on Superose 6 column. Results of SDS-PAGE demonstrated that the purity of NLGN2 was suitable for further analysis. **(B)** Cryo-EM map of NLGN2. NLGN2 forms homodimer in C2 symmetry. Two different views are shown. **(C)** Secondary structure of NLGN2 analyzed using PDBsum (54). **(D)** Superimposing NLGN2 dimer model into the cryo-EM maps. **(E)** Model of NLGN2 homodimer. SDS-PAGE, sodium dodecyl sulfate–polyacrylamide gel electrophoresis; cryo-EM, cryogenic electron microscopy.

**Figure 3:**
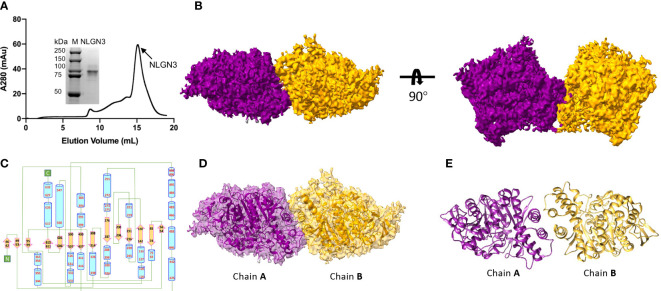
Overall structure of NLGN3. **(A)** Preparation of NLGN3. NLGN3 was expressed in HEK293f cells, purified *via* affinity chromatography and gel filtration, and eluted at about 15.1 ml on Superose 6 column. Results of SDS-PAGE demonstrated that the purity of NLGN2 was suitable for further analysis. **(B)** Cryo-EM map of NLGN3. NLGN3 forms homodimer in C2 symmetry. Two different views are shown. **(C)** Secondary structure of NLGN2 analyzed using PDBsum (54). **(D)** Superimposing NLGN3 dimer model into the cryo-EM maps. **(E)** Model of NLGN3 homodimer. SDS-PAGE, sodium dodecyl sulfate–polyacrylamide gel electrophoresis; cryo-EM, cryogenic electron microscopy.

The extracellular domain of human NLGN2 full-length human NLGN3 was expressed in HEK293F cells and purified by nickel-affinity chromatography. The gel filtration chromatography results showed that the elution volume of these two NLGN proteins was similar (15.5 ml for NLGN2 and 15.1 ml for NLGN3). Both NLGN2 and NLGN3 were eluted as one peak to near homogeneity, which was further demonstrated by SDS-PAGE analysis (**Figures 2A**, **3A**). They could form a homodimer in solution, and the peak fraction was subjected to cryo-EM sample preparation. These structures were solved with resolutions of 3.5 and 3.9 Å (**Figures 2B**, **3B**, **Supplementary Figures 1, 2** and **Supplementary Table 1**).

### Overall structures of NLGN2 and NLGN3

The NLGN structures were highly conserved in both human NLGN2 and human NLGN3 dimer forms as two symmetrical elliptical spheres, and the interface comprises a four-helix bundle including two helices from each protomer (**Figures 2B–E**, **3B–E**). For NLGN2 protomer, it contains an α/β hydrolase fold, a 13-stranded central curved β-sheet surrounded by 22 α-helices (**Figure 2C**, **Supplementary Figure 3A**). The interface area between NLGN2 dimer is about 744 Å^2^, which consisted of interacting residues (E429, H607, M434, F433, A599, Q592, L604, A439, W438, and Q596), among which E429 and H607 form salt bridges, and other residues mainly contribute hydrophobic interactions (**Supplementary Figure 3B**).

Although the NLGN3 was used at full length, the size-exclusion chromatography and SDS-PAGE analysis showed that the molecular weight of NLGN3 was similar to NLGN2 ectodomain (**Figure 3A**), with NLGN3 slightly earlier than NLGN2. There are two possibilities: one was that the full length of NLGN3 was degraded, and the other was that the C-terminal of NLGN3 was very flexible and hard to see. As the C-terminal flag tag was used for purification and NLGN3 can be purified successfully, it is possible that the C-terminal of NLGN3 was too flexible to be seen in our case. For modeled human NLGN3 dimer, the protomer consists of an α/β hydrolase fold, a 14-stranded central curved β-sheet surrounded by 25 α-helices (**Figure 3C**, **Supplementary Figure 4A**). The interface area is about 789 Å^2^, contributed by interface residues including H615, T619, F623, L627, W461, A462, F456, and A622 (**Supplementary Figure 4B**).

### Structural comparison of human NLGN2 and NLGN3

**Figure 4:**
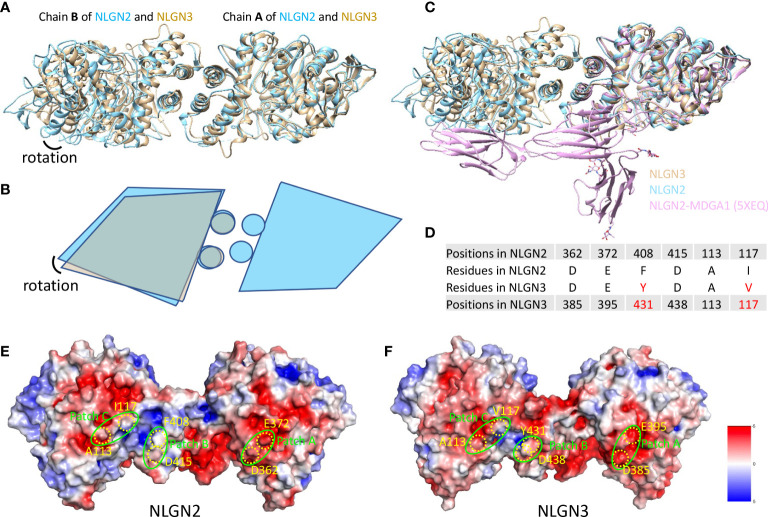
Comparison of NLGN2 and NLGN3 structures. **(A)** Structural alignment of NLGN2 and NLGN3 with chain A as reference. Chain B of NLGN2 and NLGN3 has a rotation relative to each other. **(B)** Schematic diagram showing the relative rotation of chain B as shown in panel **(A)**. **(C)** Structural alignment of NLGN2, NLGN3, and NLGN2–MDGA1 complex (PDB ID: 5XEQ). **(D)** Key residues for human NLGN2–MDGA1 interactions identified and confirmed previously (40). Electrostatic potential distributions of NLGN2 **(E)** and NLGN3 **(F)** calculated using APBS Electrostatics plugin (56) in PyMol (https://pymol.org/2/). Patches A–C critical for NLGN2–MDGA1 interaction are shown in green circles (40). Key residues shown in panel D were mapped and shown in yellow color. Unit of electrostatic potential is kT e^−1^.

Human NLGN2 and NLGN3 have distinct roles in synapse development and plasticity (55), and the development of ASD as well. To explore the potential mechanism and structural basis of their differences, we compared the structures of NLGN2 and NLGN3. The overall structure of NLGN2 and NLGN3 protomers is very similar, with root-mean-square deviation (RMSD) of 1.518 Å over 522 Cα atoms. However, when chain A from NLGN2 and NLGN3 was superimposed, the orientation of chain B from NLGN3 has a rotation relative to that from NLGN2, indicating the distinct arrangement adopted by different NLGNs (**Figures 4A, B**).

MDGAs have been reported as interacting partners of NLGNs to regulate the recognition between NLGNs and NRXNs (29, 40). X-ray crystallography structural analysis of numerous of MDGAs/neuroligin complexes provides a structural basis for understanding the role of this complex in synapses. These studies suggested that neither MDGA1 nor MDGA2 can bind to NLGN2, but not NLGN1 or NLGN3. However, there is no direct evidence to reveal the reasons for this phenomenon, especially at the molecular level. A previous study has determined the complex structure of human NLGN2 with human MDGA1 Ig1-3 domain with PDB ID 5XEQ (40); we thus also compare the NLGN2/NLGN3 structure with 5XEQ. The structural alignment shows that the relative rotation of chain B may affect the interaction between NLGN2 and MDGA1 (**Figure 4C**). To further explore more details, sequence alignment was performed using NLGNs from different species (**Supplementary Figure 5**), and the identified residues critical for human NLGN2–MDGA1 interactions were extracted and listed in **Figure 4D**. The result showed that although some residues are identical across species (D362, E372, and D415 from human NLGN2), there are some different residues in the interface (F408 and I117 from human NLGN2; A113 is identical in NLGN2 and NLGN3 but not in NLGN1). The electrostatic potential distributions of NLGN2 and NLGN3 were also calculated using APBS plugin in PyMol (56) and shown in **Figures 4E, F**. The interacting residues were mapped to the previously identified patches A–C (40). The results intuitively showed a slight difference in patch B, where F408 of NLGN2 was substituted by Y431 in NLGN3. To summarize, the differences in interface residues and distinct dimer arrangement may contribute to the different roles of NLGN2 and NLGN3.

## Discussion

Recent findings have studied the link between ASD to both type 1 and type 2 diabetes (57–59), while the results are complicated and make it hard to draw a credible conclusion. The studies based on Northern California ASD patients showed that there is a significantly increased prevalence of diabetes in ASD patients (60). However, studies based on Illinois, New York, and Texas ASD patients showed reduced prevalence (61). Also, limited by the sample sizes, researchers can only conclude that there may be an association between gestational diabetes and ASD (59). However, a convincing connection was built between maternal diabetes and ASD in a rat model (9). In rats with diabetes, transient hyperglycemia induces persistent reactive oxygen species (ROS) generation and oxidative stress-mediated histone methylation, further suppressing SOD2 expression, which was the major cause of ASD in rat offspring (9). Neuroligins, known as postsynaptic cell adhesion proteins, had been shown for decades to be related to oxidative stress regulation (62). Neuroligin-deficient animals are hypersensitive to oxidative stress and have sensory processing deficits (62). In the hypothesis of Hunter et al., neuroligins regulated ROS generation in neuronal and glial cells, and mutants in neuroligins disrupted the redox homeostasis, which might be a potential mechanism for neuroligin defect that induced ASD. This hypothesis was supported by a recent publication that showed that lutein feeding restores neuroligin expression and redox homeostasis, further rescuing neuroligin-mediated neurodevelopmental defects (63). In our studies, the expression levels of NLGN2 and NLGN3 were elevated in the maternal diabetes-related streptozotocin-induced ASD mouse model, which may be a response to the increased oxidative stress (9).

The development of ASD is closely related with neuron–neuron communications (64). Synaptic adhesion molecules mediate the communications between presynaptic and postsynaptic neurons and play critical roles in synapse development and plasticity (65). Among them, NLGNs and NRXNs are two important interacting partners across the synapse (19). Each of them has several members with different distributions and splicing isoforms (66, 67). The combination of NLGNs and NRXNs results in different affinities across the synapse, which contributes to its plasticity regulation (19). Moreover, other interacting partners such as MDGAs may also be involved in the regulation of NLGN-NRXN interactions. MDGAs may selectively interact with different NLGNs and eventually regulate the differentiation of synapses (39–41, 68). In this study, the cryo-EM structures of NLGN2 and NLGN3 were determined using cryo-EM techniques. The structures of neuroligins are highly conserved. The extracellular domain of NLGN2 is a cholinesterase-like domain, which might be required for synapse-specific functions (52). Structural comparisons show that two chains of their homodimers show distinct orientations, although their protomers adopt a similar assembly. The functional difference between NLGN2 and NLGN3 indicated that their small structural differences may cause a huge functional gap. Our results showed that the differences in interface area and orientation are the small structural differences between NLGN2 and NLGN3, which possibly confer the ability of NLGN2 to determine inhibitory synaptic transmission in neurons (23) and confer the ability of NLGN3 to control AMPAR-mediated basal excitatory transmission (69). Taken together with the previously reported complex structure of NLGN2 and MDGA1 (40), it is possible that the different orientations of NLGNs may also affect their interactions with MDGAs. This may provide one explanation for why MGDAs selectively interact with different NLGNs (39, 41, 68).

Several disease-related mutations have been reported for neuroligins, including R55G, V72X, K82Q, N236S, R451C, R471C, P534S, R617W, T659N, L721F, and T812S in NLGN3 (**Supplementary Figure 6A**, retrieved from UniProt ID Q9NZ94). Among them, R451C is a well-known mutation that is closely related to the development of ASD (70) and has recently been found to enhance the gain of function in excitatory synaptic transmission (71). This residue locates in the central helix of NLGN3 (**Supplementary Figures 6A, B**), and the mutation may affect the intracellular traffic and membrane localization of NLGN3. Knock-in of NLGN3^R451C^ has also been developed as one of the ASD mouse models (72). Sequence alignment shows that NLGN2 also has the corresponding arginine residue at position 428 in a conserved region (**Supplementary Figures 5**, **6C**). Structural superimposition demonstrates that R451 from NLGN3 and R428 from NLGN2 adopt similar conformations (**Supplementary Figure 6B**). Interestingly, only R451C from NLGN3 was reported to be related to ASD, but not R428 from NLGN2. This might be because of the subtle differences between NLGN2 and NLGN3, which still need further exploration.

## Data availability statement

The datasets presented in this study can be found in online repositories. The names of the repository/repositories and accession number(s) can be found below: http://www.wwpdb.org/, 8GS3 http://www.wwpdb.org/, 8GS4.

## Ethics statement

The animal study was reviewed and approved by Animal Welfare and Ethics Committee of Southern University of Science and Technology.

## Author contributions

ZZ, MH, JL, HZ, and ZL designed and supervised the whole project. MZ, HO, and ZL performed bioinformatics studies. ZZ, MH, and HZ prepared the samples, processed the cryo-EM data, built the model, and performed the structural analysis of NLGN2 and NLGN3. ZZ, MH, HZ, ZL, and HO prepared the manuscript. JL gave the final approval of the manuscript. All authors proofread the manuscript.

## Funding

This work was mainly supported by the Shenzhen Science and Technology Innovation Committee (No. JCYJ20200109150700942). Other funding sources include the Key-Area Research and Development Program of Guangdong Province (2019B030335001), the Shenzhen Fund for Guangdong Provincial High Level Clinical Key Specialties (No. SZGSP013), the Shenzhen Key Medical Discipline Construction Fund (No. SZXK042), the National Natural Science Foundation of China (No. 31900046, No. 82201315, No. 81972085, and No. 82172465), and the Guangdong Provincial Key Laboratory of Advanced Biomaterials (2022B1212010003). Shenzhen Clinical Research Center for Mental Disorders (No. 20210617155253001)

## Acknowledgments

We thank the Cryo-EM Center of the Southern University of Science and Technology for data collection and the HPC-Service Station. We are grateful for the assistance of the SUSTech Core Research Facilities.

## Conflict of interest

The authors declare that the research was conducted in the absence of any commercial or financial relationships that could be construed as a potential conflict of interest.

## Publisher’s note

All claims expressed in this article are solely those of the authors and do not necessarily represent those of their affiliated organizations, or those of the publisher, the editors and the reviewers. Any product that may be evaluated in this article, or claim that may be made by its manufacturer, is not guaranteed or endorsed by the publisher.

## Supplementary material

Supplementary Figure 1 Workflow of structural determination for NLGN2. Data of NLGN2 were collected using Titan krios. Raw micrographs were subjected to beam-induced motion collection and contrast transfer function (CTF) estimation. NLGN2 particles were then boxed, extracted and subjected to 2D classification, initial model building and 3D classification. Good classes were then used for final map reconstruction. The overall resolution of NLGN2 was determined to be 3.5 Å according to Fourier Shell Correlation (FSC) with 0.143 criteria.

Supplementary Figure 2 Workflow of structural determination for NLGN3. Data of NLGN3 were collected using Titan krios. Raw micrographs were subjected to beam-induced motion collection and contrast transfer function (CTF) estimation. NLGN3 particles were then boxed, extracted and subjected to 2D classification, initial model building and 3D classification. Good classes were then used for final map reconstruction. The overall resolution of NLGN3 was determined to be 3.9 Å according to Fourier Shell Correlation (FSC) with 0.143 criteria.

Supplementary Figure 3 Structural analysis of NLGN2. **(A)** Wiring diagram to show secondary structure of NLGN2. **(B)** Identified residues in the protomer-protomer interface import for NLGN2 dimerization. Figures were prepared in PDBsum (54).

Supplementary Figure 4 Structural analysis of NLGN3. **(A)** Wiring diagram to show secondary structure of NLGN3. **(B)** Identified residues in the protomer-protomer interface import for NLGN3 dimerization. Figures were prepared in PDBsum (54).

Supplementary Figure 5 Sequence alignment of NLGNs. Amino acid sequences of different NLGNs (NLGN1, NLGN2 and NLGN3) from different species (human, mouse and rat) were extracted from Uniprot (https://www.uniprot.org/) and subjected to multiple sequence alignment using Clustal Omega (73). The alignment file and NLGN2 structure were further used as input of ESPript webserver (74) to map the secondary structure.

Supplementary Figure 6 Disease-related mutations of NLGNs. **(A)** Mapping of the mutation sites in NLGN3 cryo-EM structures. Those sites include R55, V72, K82, N236, R451, R471, P534 and R617 from NLGN3. **(B)** Structural superimposition of R428 from NLGN2 and R451 from NLGN3. **(C)** Sequence alignment of human NLGN2 and human NLGN3 in the conserved arginine site regions (R428 of NLGN2 and R451 of NLGN3).

Supplementary Table 1 Summary of Cryo-EM data collection, data processing, and structure refinement.

Supplementary Table 2 Primers used in real-time quantitative PCR
