# Supplementary figures and images for: Expression and structural analysis of human neuroligin 2 and neuroligin 3 implicated in autism spectrum disorders
Source: Front Endocrinol (Lausanne). 2022 Nov 21;13:1067529. doi: 10.3389/fendo.2022.1067529 (PMC9719943; doi:10.3389/fendo.2022.1067529)

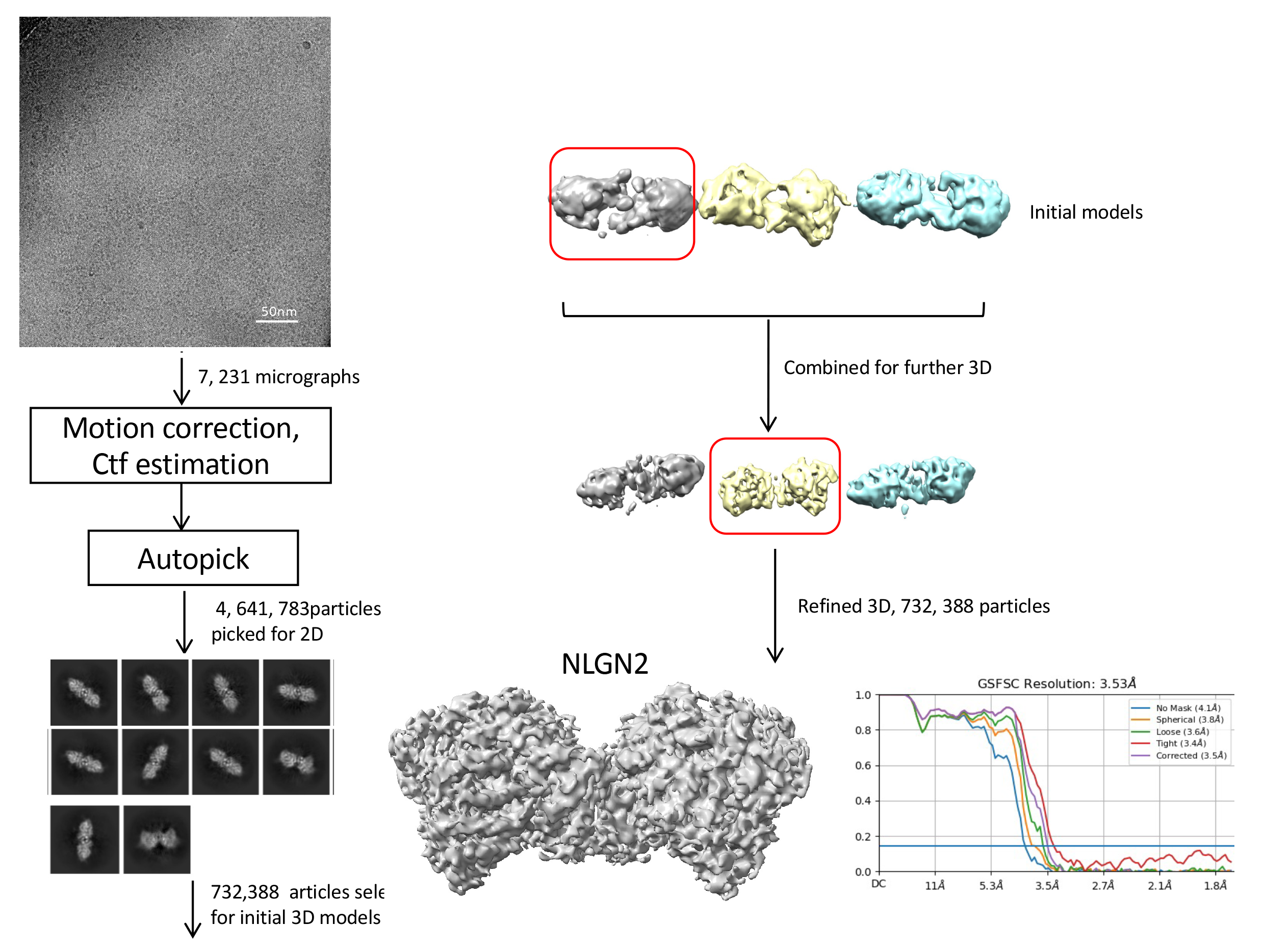

Supplement: Supplementary Figure 1 — Workflow of structural determination for NLGN2. Data of NLGN2 were collected using Titan krios. Raw micrographs were subjected to beam-induced motion collection and contrast transfer function (CTF) estimation. NLGN2 particles were then boxed, extracted and subjected to 2D classification, initial model building and 3D classification. Good classes were then used for final map reconstruction. The overall resolution of NLGN2 was determined to be 3.5 Å according to Fourier Shell Correlation (FSC) with 0.143 criteria. [file Image_1.tif]

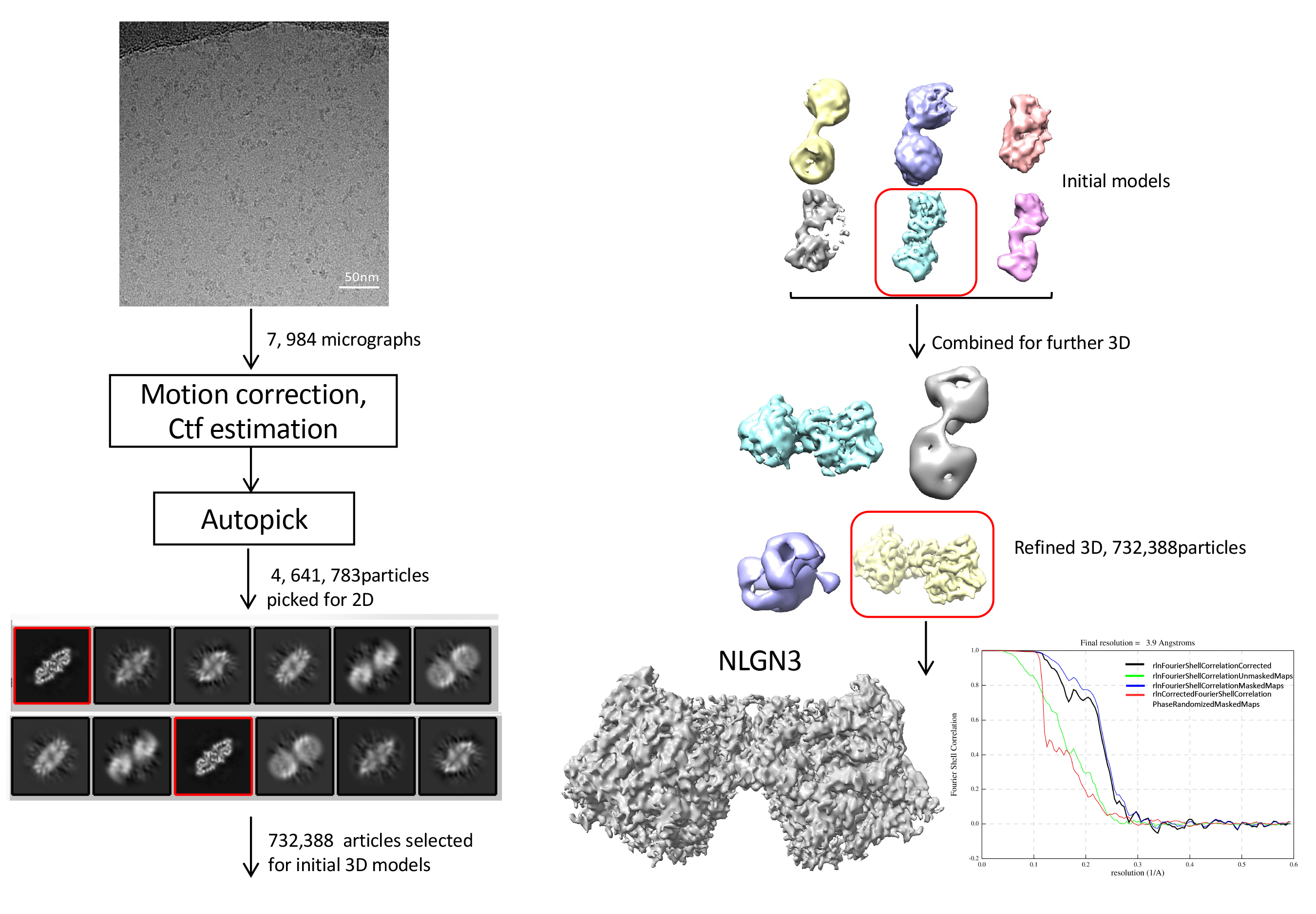

Supplement: Supplementary Figure 2 — Workflow of structural determination for NLGN3. Data of NLGN3 were collected using Titan krios. Raw micrographs were subjected to beam-induced motion collection and contrast transfer function (CTF) estimation. NLGN3 particles were then boxed, extracted and subjected to 2D classification, initial model building and 3D classification. Good classes were then used for final map reconstruction. The overall resolution of NLGN3 was determined to be 3.9 Å according to Fourier Shell Correlation (FSC) with 0.143 criteria. [file Image_2.tif]

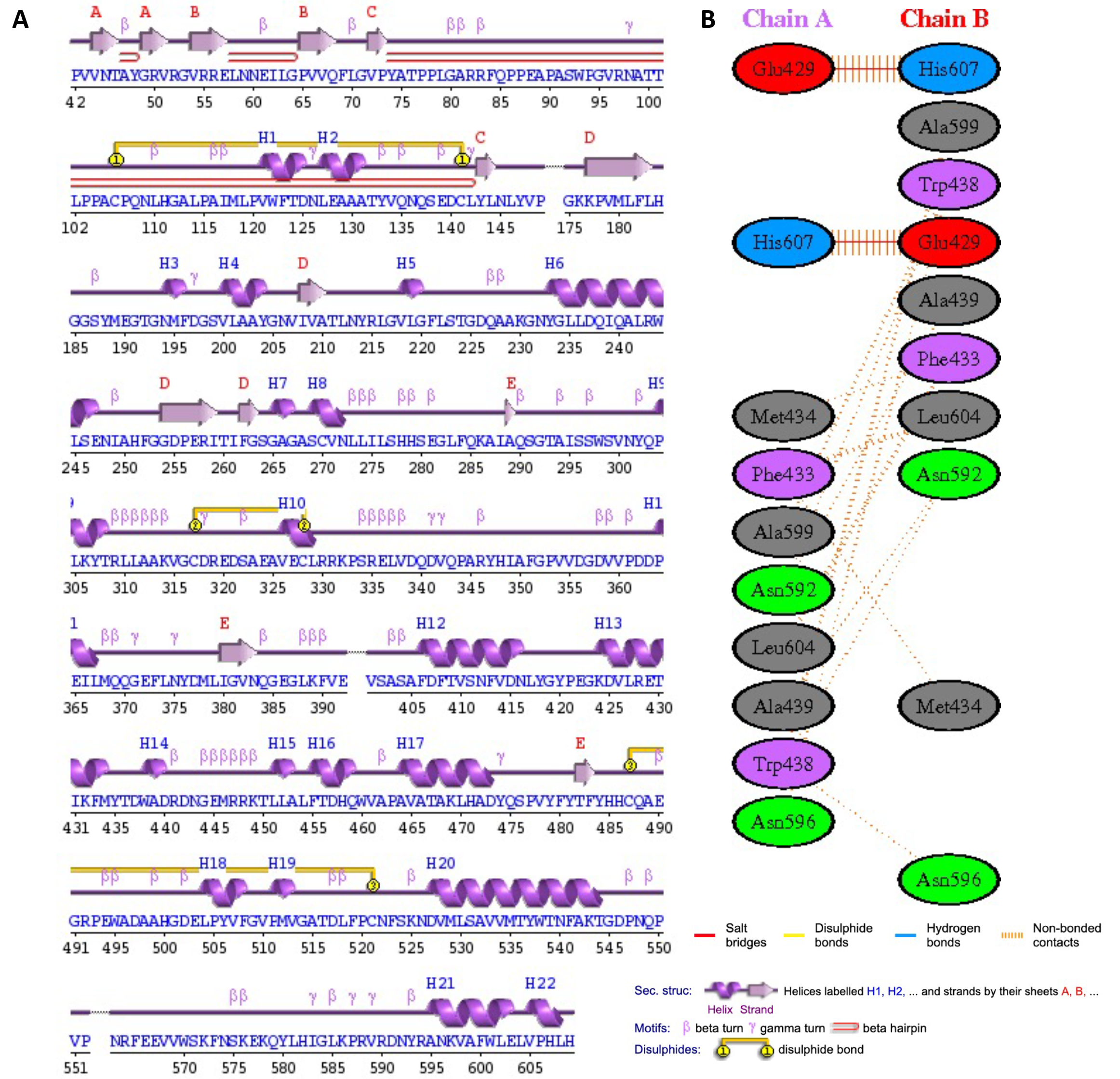

Supplement: Supplementary Figure 3 — Structural analysis of NLGN2. (A) Wiring diagram to show secondary structure of NLGN2. (B) Identified residues in the protomer-protomer interface import for NLGN2 dimerization. Figures were prepared in PDBsum (54). [file Image_3.tif]

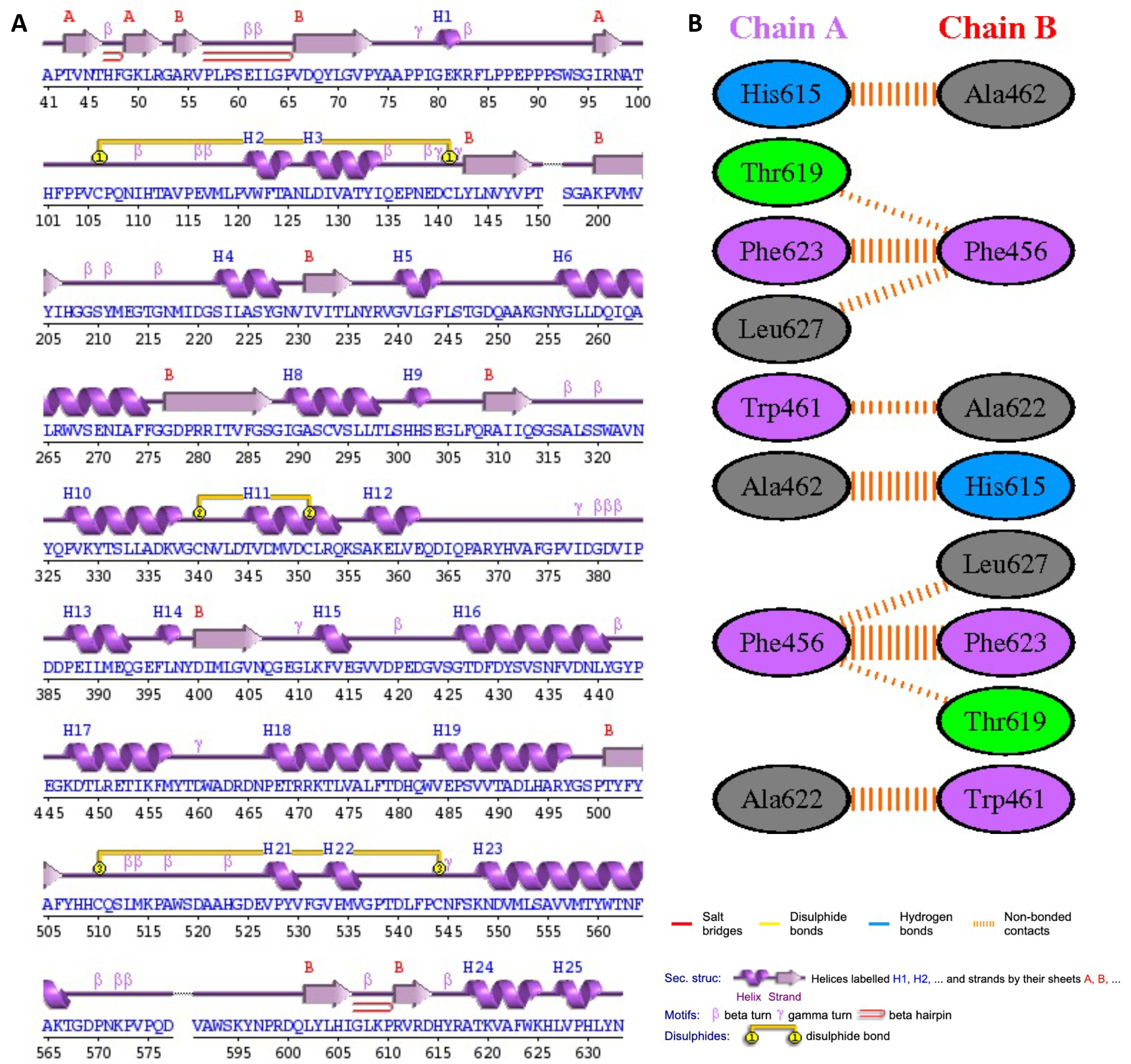

Supplement: Supplementary Figure 4 — Structural analysis of NLGN3. (A) Wiring diagram to show secondary structure of NLGN3. (B) Identified residues in the protomer-protomer interface import for NLGN3 dimerization. Figures were prepared in PDBsum (54). [file Image_4.tif]

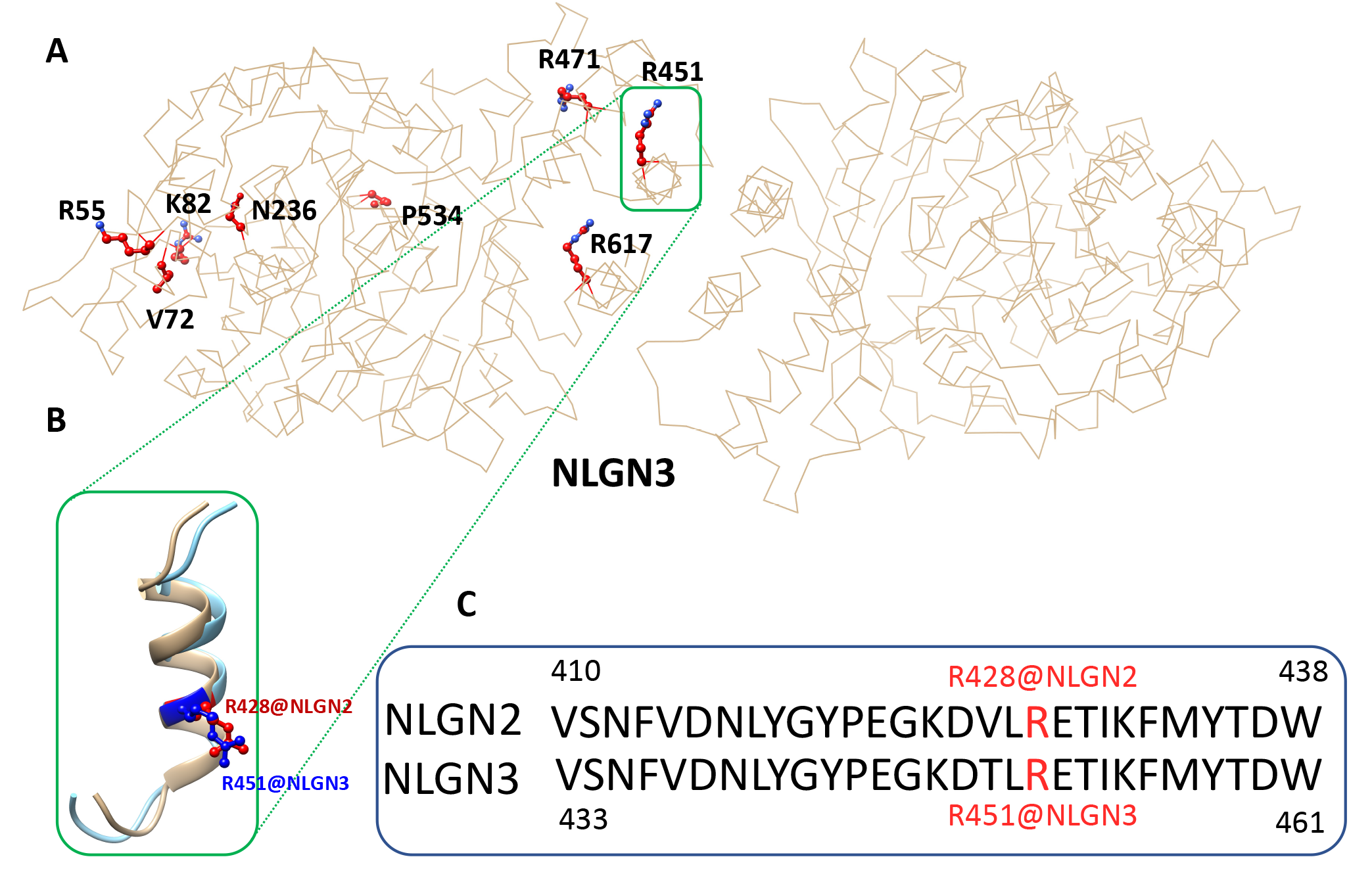

Supplement: Supplementary Figure 6 — Disease-related mutations of NLGNs. (A) Mapping of the mutation sites in NLGN3 cryo-EM structures. Those sites include R55, V72, K82, N236, R451, R471, P534 and R617 from NLGN3. (B) Structural superimposition of R428 from NLGN2 and R451 from NLGN3. (C) Sequence alignment of human NLGN2 and human NLGN3 in the conserved arginine site regions (R428 of NLGN2 and R451 of NLGN3). [file Image_6.tiff]
