# Supplementary material for: Expression and structural analysis of human neuroligin 2 and neuroligin 3 implicated in autism spectrum disorders
Source: Front Endocrinol (Lausanne). 2022 Nov 21;13:1067529. doi: 10.3389/fendo.2022.1067529 (PMC9719943; doi:10.3389/fendo.2022.1067529)
Supplement: Supplementary Table 1 — Summary of Cryo-EM data collection, data processing, and structure refinement. [file Table_1.docx]

**Supplementary Table S1. Summary of Cryo-EM data collection, data processing, and structure refinement.**

| Dataset | **NLGN3** | **NLGN2** |
| --- | --- | --- |
| EMDB and PDB No. | EMD-34219, 8GS3 | EMD-34220, 8GS4 |
| **Data collection** |  |  |
| EM equipment | Titan krios | Titan krios |
| Voltage (kV) | 300 | 300 |
| Detector | Gatan K2 Summit | Gatan K3 Summit |
| Energy filter | Gatan GIF, 20 eV slit | Gatan GIF, 20 eV slit |
| Pixel size (Å) | 0.842 | 0.92 |
| Total Electron dose (e^-^Å^-2^) | 50 | 50 |
| Defocus range (µm) | -1.5~-2.5 | -1.5~-2.5 |
| **3D Reconstruction** |  |  |
| Software | Relion | cryoSPARC |
| Number of micrographs | 7,984 | 4,021 |
| Final particles | 255,939 | 192,341 |
| Symmetry | C2 | C2 |
| Final resolution (Å) | 3.9 | 3.5 |
| Map sharpening B-factor (Å²) | -182 | -227 |
| **Refinement** |  |  |
| Software | Phenix | Phenix |
| **Model composition** |  |  |
| Protein residues | 1066 | 1050 |
| ligand | 0 | 0 |
| **R.M.S. deviations** |  |  |
| Bonds length (Å) | 0.009 | 0.007 |
| Bonds Angle (˚) | 0.949 | 0.934 |
| MolProbity score | 2.28 | 2.34 |
| Clash Score | 17.8 | 14.48 |
| Rotamer outliers | 0 | 0 |
| **Ramachandran plot statistics (%)** |  |  |
| Preferred | 90.62 | 84.24 |
| Allowed | 9.38 | 15.76 |
| Outliers | 0 | 0 |
