# Supplementary material for: Expression and structural analysis of human neuroligin 2 and neuroligin 3 implicated in autism spectrum disorders
Source: Front Endocrinol (Lausanne). 2022 Nov 21;13:1067529. doi: 10.3389/fendo.2022.1067529 (PMC9719943; doi:10.3389/fendo.2022.1067529)
Supplement: Supplementary Table 2 — Primers used in real-time quantitative PCR [file Table_2.docx]

**Supplementary Table S2. Primers used in real-time quantitative PCR**

| **Gene** | **Species** | **Analysis** | **Forward primer (5'→3')** | **Reverse primer (5'→3')** |
| --- | --- | --- | --- | --- |
| *NLGN2* | Mouse | mRNA | TGTCATGCTCAGCGCAGTAG | GGTTTCAAGCCTATGTGCAGAT |
| *NLGN3* | Mouse | mRNA | CCCTGGGCTTCCTCAGTTTG | GGCAATGGTACTCTGGCACC |
| *β-actin* | Mouse | mRNA | GTGACGTTGACATCCGTAAAGA | GCCGGACTCATCGTACTCC |
